# Comparison of different smartphone cameras to evaluate conjunctival hyperaemia in normal subjects

**DOI:** 10.1038/s41598-018-37925-5

**Published:** 2019-02-04

**Authors:** Carles Otero, Nery García-Porta, Juan Tabernero, Shahina Pardhan

**Affiliations:** Vision and Eye Research Unit, School of Medicine, Anglia Ruskin University, Cambridge, UK

## Abstract

Despite the significant advantages that smartphones’ cameras can provide in teleophthalmology and artificial intelligence applications, their use as black-box systems for clinical data acquisition, without adequate information of the quality of photographs can compromise data accuracy. The aim of this study is to compare the objective and subjective quantification of conjunctival redness in images obtained with calibrated and non-calibrated cameras, in different lighting conditions and optical magnifications. One hundred ninety-two pictures of the eye were taken in 4 subjects using 3 smartphone cameras{Bq, Iphone, Nexus}, 2 lighting levels{high 815 lx, low 122 lx} and 2 magnification levels{high 10x, low 6x}. Images were duplicated: one set was white balanced and color corrected (calibrated) and the other was left as it was. Each image was subjective and objectively evaluated. There were no significant differences in subjective evaluation in any of the conditions whereas many statistically significant main effects and interaction effects were shown for all the objective metrics. The clinician’s evaluation was not affected by different cameras, lighting conditions or optical magnifications, demonstrating the effectiveness of the human eye’s color constancy properties. However, calibration of a smartphone’s camera is essential when extracting objective data from images.

## Introduction

Telemedicine has already become an emergent field of interest in image-based medical specialty including ophthalmology^1,2^. Image capture is likely the most important component for a tele-ophthalmology system as poor image quality would lead to a poor diagnosis.

Recent widespread use of smart phone cameras attached to ophthalmic imaging systems, in particular to the eyepiece of the slit-lamp^3^, has enabled acquisition of high-quality images of the eye in a simple and affordable manner^2,4^. There are many studies that have used smartphones for teleophthalmology^5–9^. In 2012, it was estimated that about 81% of the US physicians had smartphones^5^. Thus, using smartphones is convenient and portable, alleviating the need to carry bulky equipment to a remote site^9^. The wireless connection of the smartphones also adds another advantage by providing easy Internet connection, which is an advantage not only for teleophthalmology but also for artificial intelligence applications where thousands of images from different institutions are stored altogether in a cloud service for further processing^10,11^. Another application that has received increasing interest is the automated and objective assessment information extracted from photographic-based methods, for example, in determining ocular redness^12–18^. An assessment of conjunctival hyperaemia with a slit-lamp biomicroscope is a vital part of any ophthalmic evaluation. Ocular redness can indicate not only certain systemic conditions^19–21^ but also ocular conditions such as anterior eye inflammation^22^, allergic and infective conjunctivitis^23^, contact lens wear^24^, meibomian gland dysfunction^25^ and dry eye disease^26^.

Despite the significant advantages that smartphones’ cameras provide, their use as black-box systems for clinical data acquisition, without adequate information of the quality of photographs would compromise data accuracy and repeatability^27^. In scientific and professional photography it is well-known that two different cameras, or even the same camera with different settings, give different images for the same scene, which are possibly different to those directly perceived by our visual system. One reason is that the responses of the camera sensors vary from one camera to another. The Red, Green and Blue (RGB) values given by any imaging system are device dependent^28^. Another reason is that the responses of the three RGB sensors of a camera are different from the responses of the retina cells, and subsequent interpretation by the brain^28,29^. In addition, it is also well-known that camera manufacturers have their own camera-specific and proprietary image processing algorithms^27^, including autofocus algorithms that try to automatically enhance the perceptual image quality of the images. It is reasonable to assume that the most frequent use of smartphones’ cameras is the autofocus mode. This fact adds lack of control and introduces uncertainty of color reproductions of clinical images obtained with different smartphones.

To our knowledge, no one has evaluated the impact of smartphone’s camera calibration in ophthalmology applications, despite the fact that smartphones are used with a slit-lamp to image, in the autofocus mode, to take images of anterior segment of the eye. Therefore, the purpose of this study is to evaluate and quantify the influence of different smartphone cameras, lighting conditions and optical magnifications on various objective and subjective quantification of eye’s redness.

## Methods

### Subjects

A total of 192 images from 4 healthy adult subjects were obtained (24 experimental conditions × 2 processing approaches × 4 subjects). Subjects had a best-corrected visual acuity equal or better than 0 logMAR (20/20) in both eyes, a mean spherical equivalent of −4.75 ± 2.50 D and no ocular pathologies. Subjects did not wear contact lenses during the experiment and none of them had anterior eye problems. The mean age ± standard deviation was 32 ± 4 years. The study was approved by the Ethics Committee of Anglia Ruskin University (Cambridge, UK), it followed the tenets of the Declaration of Helsinki and all subjects gave informed written consent.

### Examination protocol

**Figure 1:**
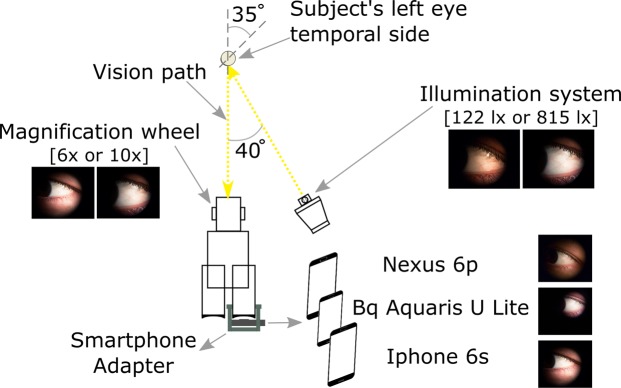
Schematic representation of the setup used to take pictures of the temporal conjunctiva of each subject’s left eye. Pictures were taken with 3 different smartphones attached to a slit lamp and with two different magnification and illumination conditions. The pictures of the eyes shown in this figure correspond to the same subject.

**Figure 2:**
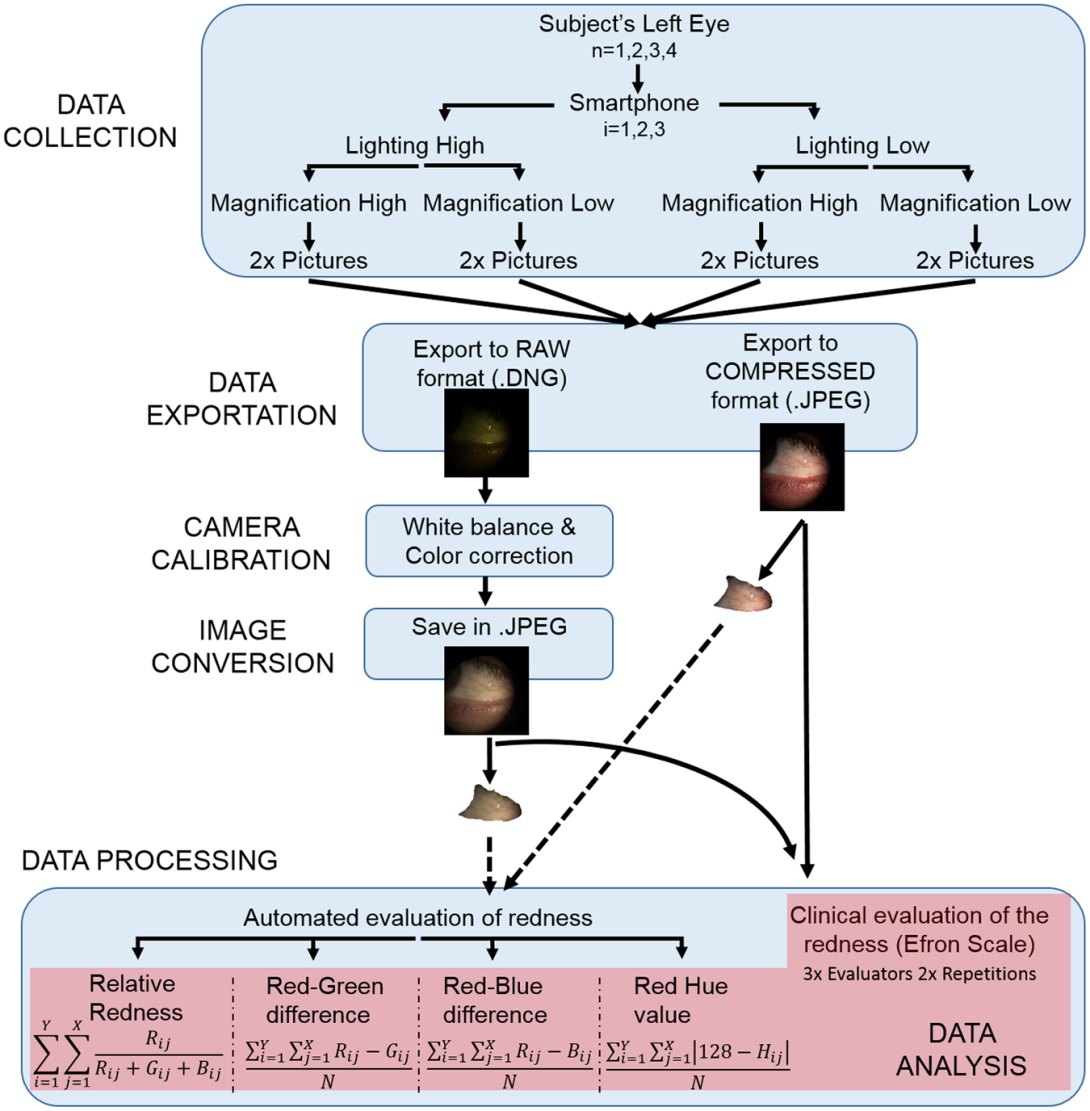
Processing steps conducted for each smartphone and eye. X and Y are the pixel coordinates in the horizontal and vertical directions. R, G and B refer to the Red, Green and Blue channels of a camera sensor. N is the total number of pixels. H is the Hue channel of an image in the HSL color space.

Pictures of the temporal side of the left eye were taken in 4 subjects under 24 experimental conditions: camera type {smartphone 1: Bq Aquaris U Lite, smartphone 2: Iphone 6 s, smartphone 3: Nexus 6p}, lighting level {high 815 lx, low 122 lx}, magnification {high 10x, low 6x}. Two images were taken in each condition with an interval of 5 s between them in which participants were required to blink and fixate again the target (Fig. 1). Randomization among all conditions was applied. For each subject, all measurements were taken in one session that took less than 20 minutes. A diagram of all the study design is shown in Fig. 2.

The camera’s specifications of each smartphone are detailed in Table 1. All pictures were taken with the application Adobe Photoshop Lightroom CC v3.5.1 (Adobe Systems Inc., USA) and exported in two formats: raw format (.DNG) and compressed format (.JPEG). The slit lamp used for the study was the SL9800 (CSO Srl, Italy) and the smartphone and the slitlamp were connected by the HookUpz 2.0 universal optics adapter (Carson Optical Inc., USA). The magnification levels (6x or 10x) chosen in the study are typically used for slit-lamp evaluation of the conjunctiva. The lighting levels (122 lx or 815 lx) were determined prior by a clinical optometrist for best clinical evaluation.

**Table 1:** Specifications of each smartphone’s camera.

Smartphone	Brand	Model	Number of Pixels	Aperture	Sensor Size	Pixel size
1	Nexus	6 P Huawei	12.3 MP	f/2.0, 29 mm	1/2.3″	1.55 um
2	BQ	Aquaris U Lite	8 MP	f/2.0	Unknown	Unknown
3	Iphone	6 s	12 MP	f/2.2, 29 mm	1/3″	1.22 um

For each image captured by the camera, two different images were obtained (i.e., the calibrated and the non-calibrated image). The calibration procedure of the raw images was performed with MATLAB 2018 (Mathworks Inc, USA) using image computation steps as explained by Akkayanak *et al*.^27^. This involved taking a picture of a color reference target (ColorChecker Passport, X-Rite Inc., USA) for each smartphone and lighting condition and then compute a color transformation matrix that maps each RGB intensity values obtained by each camera with the color ground truth. Detailed mathematical explanations can be found elsewhere^27,28^.

For subjective assessment, bulbar redness of all images were subjectively graded by three clinicians using the Efron grading scale^30^ (from 0 to 1 in steps of 0.1, being 0 normal and 4 severe) in a blinded fashion: none of the clinicians were aware which experimental condition each image corresponded to.

For objective assessment four objective redness metrics (i.e., relative redness, red-green difference, red-blue difference and red hue) were computed for each image. These metrics have been used in previous studies and are explained in detail elsewhere^31^. The images had to be manually cut leaving only the conjunctiva before the objective metric was computed, i.e., the iris, the eyelids and any artifacts such as saturated zones were not included in the computation (Fig. 2).

### Data analysis

First, the repeatability and agreement between clinicians were analyzed by means of the within-subject standard deviation and the 95% Limits of Agreement, respectively. Additionally, the repeated measures ANOVA was computed to test whether the differences between clinicians were statistically significant or not.

Second, we evaluated the correlation between the objective metrics and the subjective scores for each factor. Then, a 4-way repeated measures ANOVA was computed for the 4 objective redness metrics, and the subjective evaluation values. The four within-subjects factors were: camera type {smartphone 1: Bq Aquaris U Lite, smartphone 2: Iphone 6 s, smartphone 3: Nexus 6p}, lighting level {high 815 lx, low 122 lx}, magnification {high 10x, low 6x} and calibration {pre- and post- calibration}.

Significance was set at 0.05 and the statistical analysis was performed using MATLAB 2018 (MathWorks, Inc., USA). Normality of each variable was verified with the Shapiro-Wilk test. The Bonferroni correction was applied for pairwise comparisons. The post-hoc statistical power was computed with the free open-source G*Power 3.0.10 and a value above 0.9 was obtained for all response variables.

## Results

**Figure 3:**
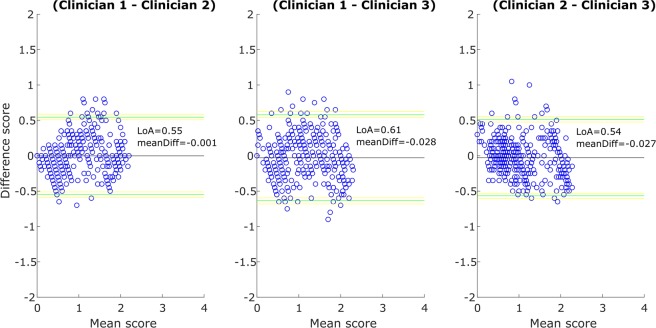
Bland and Altman plots. Agreement between clinicians. Green lines: Superior and Inferior 95% Limits of Agreement (LoA). Yellow lines: 95% confidence interval for each LoA. meanDiff.: mean score difference.

The within-subject standard deviation (repeatability) obtained for each clinician was 0.11, 0.22 and 0.27. The 95% Limits of Agreement between clinicians are shown in the Bland and Altman plots of the Fig. 3, the differences between clinicians are not statistically significant (repeated measures ANOVA, F = 2.36, p = 0.09).

Pearson correlation coefficients between each objective redness metric (i.e., Relative Redness, Red-Green difference, Red-Blue difference and Red Hue difference) and the subjective evaluation are shown in Table 2. Correlations were computed for all factors together (without grouping for factors) and also they were computed grouping according to calibration and smartphone type factors.

**Table 2:** Pearson correlation coefficients (r-values) between the objective and the subjective measures of one of the evaluators computed without grouping for factors and also grouping for calibration and smartphone.

		Correlation Subjective vs Objective			
		Relative Redness	RGdiff	RBdiff	RedHue
All factors together (without grouping)		0.43*	0.51*	0.46*	0.51*
PreCalibration	Bq	0.70*	0.85*	0.84*	0.73*
	Iphone	0.79*	0.74*	0.69*	0.73*
	Nexus	0.63*	0.35	0.34	0.61*
PostCalibration	Bq	0.59*	0.71*	0.76*	0.68*
	Iphone	0.75*	0.86*	0.75*	0.79*
	Nexus	0.61*	0.43	0.21	0.69*

*Statistically significant (p < 0.05). RGdiff.: Red-Green difference. RBdiff.: Red-Blue difference.

The results obtained for the 4-way repeated measures ANOVA applied to each objective metric and the subjective evaluation are shown in Table 3. None of the factors nor interactions were statistically significant for the subjective evaluation. On the other hand, there was a statistically significant effect of each factor (calibration, camera, lighting and magnification) for all the 4 objective metrics as well as many significant 2-factor and 3-factor interactions, in particular for the Relative Redness metric. Given that the results obtained for the 4 objective metrics are very similar and in order to disentangle all the significant effects (and keep the text concise), only the results for the Relative Redness will be analyzed further.

**Table 3:** Results of the repeated measures ANOVA with 4 within-subjects factors.

	Objective Evaluation				Subjective Evaluation
	Relative Redness	RGdiff	RBdiff	RedHue	Evaluator 1
	p-value	p-value	p-value	p-value	p-value
Calibration	0.007*	0.015*	0.035*	0.010*	0.759
Camera	0.027*	0.009*	0.004*	0.010*	0.929
Lighting	0.015*	<0.001*	<0.001*	0.020*	0.069
Magnification	0.006*	<0.001*	<0.001*	0.001*	0.116
Calibration*Camera	0.003*	0.001*	<0.001*	0.002*	0.056
Calibration*Lighting	0.027*	<0.001*	<0.001*	0.135	0.094
Camera*Lighting	0.079	0.073	0.051	0.171	0.126
Calibration*Magnification	0.006*	0.005*	0.178	0.004*	0.498
Camera*Magnification	0.013*	0.184	0.102	0.028*	0.054
Lighting*Magnification	0.965	0.699	0.827	0.288	0.168
Calibration*Camera*Lighting	0.020*	0.002*	<0.001*	0.244	0.247
Calibration*Camera*Magnification	<0.001*	0.138	0.147	<0.001*	0.167
Calibration*Lighting*Magnification	0.022*	0.728	0.966	0.720	0.301
Camera*Lighting*Magnification	0.647	0.374	0.436	0.514	0.224
Calibration*Camera*Lighting*Magnification	0.924	0.558	0.840	0.343	0.602

*Statistically significant (p < 0.05). RGdiff.: Red-Green difference. RBdiff.: Red-Blue difference.

**Figure 4:**
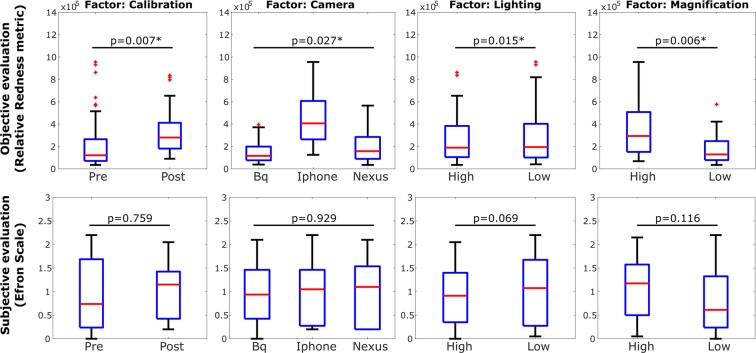
Boxplots comparing each factor obtained for the relative redness metric (first row) and the subjective evaluation of redness (seconds row). *Statistically significant (p < 0.05).

**Figure 5:**
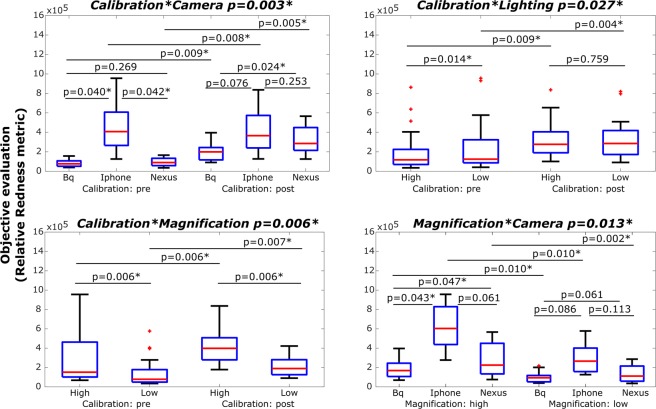
Boxplots for each statistically significant 2-way interaction found in Table 3. *Statistically significant pairwise comparisons (p < 0.05).

The main effects of the Relative Redness and the subjective evaluation are shown in Fig. 4, and all the statistically significant 2-factor interactions obtained for the Relative Redness, i.e., Calibration*Camera, Calibration*Lighting, Camera*Lighting and Calibration*Magnification, are summarized in the boxplots of Fig. 5. For completeness, despite its much more difficult interpretation, the statistically significant 3-factor interactions obtained for the Relative Redness, i.e., Calibration*Camera*Lighting, Calibration*Camera*Magnification and Calibration*Lighting*Magnification are summarized in Fig. S1 (Supplementary Fig. S1).

## Discussion

In a clinical setting, the ophthalmic evaluation using the slit-lamp is perhaps one of the most often methods conducted in every patient. Commercial slit-lamps offer a wide range of light intensities as well as magnifications, and each clinician can use the combination of both parameters that best fits each particular case and purpose. In addition, the use of commercial digital cameras attached to a slit-lamp to image the anterior and posterior segment of the eye are becoming more popular every day in clinical settings, particularly for teleophthalmology and artificial intelligence applications^10,11^. We investigated the effect of smartphone’s camera calibration, camera’s type, slit-lamp lighting level and magnification, as well as their interactions on one specific imaging application: the objective and subjective quantification of ocular redness. Five variables were studied: 4 objective ocular redness metrics and the subjective grading score of redness according to the Efron grading scale^30^.

The repeatability of all three evaluators (0.11, 0.22 and 0.27 units) is consistent with previous studies. Chong *et al*.^32^ evaluated the repeatability of 5 evaluators using three different anterior segment clinical grading scales: (1) verbal descriptors scale, (2) photographic matching scale, and (3) continuous matching scale. They reported grading scales to be repeatable and obtained within-subject standard deviations for the different evaluators between 0.11 and 0.18 units. Murphy *et al*.^33^ found inter-observer 95% limits of agreement for bulbar redness of 0.38 units. Our values are slightly higher (0.55, 0.61, 0.54 units), it is possible that these differences are due a much lower sample size and a different grading scale, i.e., Murphy *et al*. used the Cornea and Contact Lens Research Unit (CCRLU) grading scale in 20 images only.

The degree of relationship between the objective and subjective evaluations, examined with the Pearson correlation coefficient and considering all factors together, ranged between 0.43 and 0.51 for 4 different objective metrics. The 4 objective metrics gave similar correlation coefficients, i.e., none of the objective metrics outperformed over the others, however, the correlation coefficients were in general smaller in comparison to Papas’ study^31^ who reported correlations of 0.70, 0.70, 0.72 and 0.41 for Relative Redness, Red-Green difference, Red-Blue difference and Red Hue value metrics, respectively. Our data suggests that the slightly weaker correlations may be influenced by other factors that we have studied which are calibration, camera’s type, lighting level and magnification which show up as important covariates in the objective quantification of ocular redness. This is indeed corroborated by the 4-way repeated measures ANOVA applied to the objective variables, and the significant main effects of calibration, camera, lighting and magnification, and also several strong 2-factor and 3-factor interactions (Table 3).

It is remarkable to note none of the factors nor their interactions significantly affected the subjective evaluations, which directly relates to the inherent property of the human eye of color constancy, i.e., the perceived color of a surface remains constant despite changes in the conditions of illumination^34^.

With regards to magnification factor, it is somewhat not surprising that there were significant differences between different magnifications (6x and 10x). The Relative Redness values are directly proportional to the area being considered, therefore, when magnifying an image from 6x to 10x, the objective metrics values obtained for the same eye under the same conditions are increased simply because a large area is considered. In this study, each image was a different size as the sensor size was different in each smartphone and also because the region of interest was manually selected to find the largest conjunctival area possible. However, this is not the only reason that explains the differences between magnification levels. It is well-known that microscopes (and hence magnification) introduce strong color distortions particularly in the periphery of the visual field^28^, therefore, when magnifying an image in a commercial slit-lamp, the same features of an eye are imaged further from the center, into a region with a stronger color distortion.

Exploring the differences between low and high light intensities, our results suggest that these differences are mainly derived because of their strong interaction with magnification, camera’s type and whether images are calibrated or not. Given that slit-lamps do not provide a homogeneous light field and the fact that each camera can have a different response to light intensity in each red, green and blue color channel^28^ (RGB), this would undoubtedly lead to a different color reproduction of an image of the same eye. This difference in color is partially solved by calibrating for each camera and lighting level.

The significant differences between the type of smartphones when computing relative redness are possibly not only because of different hardware specifications but because each picture was taken in autofocus mode. This introduces a different internal image enhancement strategy which is designed by the manufacturer to provide the most perceptually realistic image.

This is the first time that the performance of 3 different smartphone cameras were evaluated in the context of a clinical application, and it is quite surprising such large differences found for the Iphone in comparison with the other two smartphones. To analyze this further, we computed the correlation between the relative redness metric and the subjective evaluation of each image, according to each smartphone for uncalibrated images. Pearson correlation coefficient between the Iphone and subjective evaluation was 0.79, whereas for the Bq and the Nexus these values were, respectively, 0.70 and 0.63. In all 3 cases the Pearson correlations were statistically significant (p < 0.05). It is interesting to see that after controlling for the camera’s type and calibration factors, the correlation coefficients increased and were very similar to those obtained by Papas^31^.

Our study highlights the importance of controlling for camera’s type and lighting levels when extracting objective data. Consequently, it provides further support to the fact that the correlation between objective data and subjective clinical scores are strongly influenced by these factors too. The results of this study could also potentially be applied to other cameras, including professional slit-lamp cameras, as long as it is possible to export the raw data images. The key differences between a professional slit-lamp camera and a smartphone camera (attached to the eyepiece of a slit-lamp) are: (1) the field of view of the professional slit-lamp camera is optimized to match the field of view seen through the eyepiece by the observer. This does not occur in the case of smartphone cameras attached to the slit-lamp. (2) A professional slit-lamp camera does not require an autofocus algorithm as it relies on the observer’s choice of focus. Having these two points in mind, significant differences could be expected between pictures taken with different cameras if they are not calibrated and they have a different pixel size, sensor size, sensitivity and optics.

The application of white balance and color correction to each image obtained under certain illumination conditions and with one specific camera is a standard procedure to obtain the color ground truth of the scene being photographed^27^. In this study it is shown that calibration has a strong impact on objective ocular redness measurement, as it strongly interacts with all the other factors. Our results show that any differences between lighting levels and camera’s types are significantly minimized after the cameras are calibrated. It is noteworthy that is was not possible to obtain a perfect match of relative redness values between cameras or light levels even after calibration. Ideally, if the calibration were perfect, no significant differences between smartphones and lighting levels after camera calibration would have been obtained, but, the calibration through a slit-lamp was affected by the strong inhomogeneous light field introduced by the optical system. More elaborated calibration procedures (which are more difficult to implement them in a clinical environment) could possibly be applied to improve the calibration’s accuracy, particularly when photographing through a microscope (such as the slit-lamp)^28^, however these were out of the scope of this study.

On comparing the influence of camera calibration on the subjective clinical evaluation of redness, our results showed no significant effects in different experimental conditions. This is explained by the color constancy property of human vision. However, if objective data is to be extracted and compared with other images obtained under different experimental conditions (i.e., other sensors, illumination or magnification types), camera calibration becomes an essential thing to do.

In conclusion, smartphone’s camera calibration is essential when comparing images of the eye obtained with different smartphones and/or lighting levels by means of objective metrics. The clinical evaluation of eye’s images is not affected by calibration, type of smartphone camera and/or lighting level thanks to the human eye property of color constancy. Future studies should include diseased eyes with higher redness scores.

## Supplementary information

Figure 1S

## References

[1] Kumar, S., Yogesan, K., Goldschmidt, L. & Cuadros, J. *Teleophthalmology*. (Springer-Verlag, 2006).

[2] Kumar S, Yogesan K, Constable I (2009). Telemedical diagnosis of anterior segment eye diseases: validation of digital slit-lamp still images. Eye.

[3] Barsam A, Bhogal M, Morris S, Little B (2010). Anterior segment slitlamp photography using the iPhone. J. Cart. Refract. Surg..

[4] Ye Y (2013). Resolution of slit-lamp microscopy photography using various cameras. Eye Contact Lens.

[5] Chhablani J, Kaja S, Shah V (2012). Smartphones in ophthalmology. Indian J Ophthalmol.

[6] Stanzel B, Meyer C (2012). Smartphones in ophthalmology: Relief or toys for physicians [in German]. Ophthalmologe.

[7] Ciemins E, Coon P, Sorli C (2010). An analysis of data management tools for diabetes self-management: Can smart phone technology keep up?. J Diabetes Sci Technol.

[8] Kumar S, Wang E, Pokabla M, Noecker R (2012). Teleophthalmology assessment of diabetic retinopathy fundus images: Smartphone versus standard office computer workstation. Telemed J E Heal..

[9] Ye Y (2014). Global teleophthalmology with iPhones for real-time slitlamp. Eye.

[10] Gulshan V (2016). Development and Validation of a Deep Learning Algorithm for Detection of Diabetic Retinopathy in Retinal Fundus Photographs. JAMA.

[11] Rajalakshmi R, Subashini R, Mohan R, Viswanathan A (2018). Automated diabetic retinopathy detection in smartphone-based fundus photography using artifi cial intelligence. Eye.

[12] Wolffsohn, J. & Purslow, C. Clinical monitoring of ocular physiology using digital image analysis. *Contact Lens Anterior Eye* **26** (2003). 10.1016/S1367-0484(02)00062-0 16303494

[13] Wolffsohn J (2004). Incremental nature of anterior eye grading scales determined by objective image analysis. Br. J. Ophthalmol..

[14] Amparo, F., Wang, H., Emami-Naeini, P., Karimian, P. & Dana, R. The Ocular Redness Index: a novel automated method for measuring ocular injection. *Investig. Opthalmology Vis. Sci*. **54** (2013). 10.1167/iovs.13-12217 PMC3720148 23766472

[15] Guillon M, Shah D (1996). Objective measurement of contact lens-induced conjunctival redness. Optom. Vis. Sci..

[16] Sorbara L, Simpson T, Duench S, Schulze M, Fonn D (2007). Comparison of an objective method of measuring bulbar redness to the use of traditional grading scales. Contact Lens Anterior Eye.

[17] Fieguth P, Simpson T (2002). Automated measurement of bulbar redness. Investig. Ophthalmol. Vis. Sci..

[18] Downie LE, Keller PR, Vingrys AJ (2016). Assessing ocular bulbar redness: a comparison of methods. Ophthalmic Physiol. Opt..

[19] Klaassen-Broekema N, Van Bijsterveld O (1993). Diffuse and focal hyperaemia of the outer eye in patients with chronic renal failure. Int Ophthalmol.

[20] Cheung A, Ramanujam S, Greer D, Kumagai L, Aoki T (2001). Microvascular abnormalities in the bulbar conjunctiva of patients with type 2 diabetes mellitus. Endocr Pr..

[21] Owen C, Fitzke F, Woodward E (1996). A new computer assisted objective method for quantifying vascular changes of the bulbar conjunctivae. Ophthalmic Physiol. Opt..

[22] Leibowitz H (2000). The red eye. N Engl J Med.

[23] Friedlaender M (2004). Objective measurement of allergic reactions in the eye. Curr Opin Allergy Clin Immunol.

[24] Efron N, Chaudry A (2007). Grading static versus dynamic images of contact lens complications. Clin. Exp. Optom..

[25] Suzuki T (2012). Meibomitis-related keratoconjunctivitis: implications and clinical significance of meibomian gland inflamation. Cornea.

[26] Rodriguez JJD (2013). Automated grading system for evaluation of ocular redness associated with dry eye. Clin Ophthalmol.

[27] Akkaynak D (2014). Use of commercial off-the-shelf digital cameras for scientific data acquisition and scene-specific color calibration. J Opt Soc Am A.

[28] Charrière R, Hébert M, Trémeau A, Destouches N (2013). Color calibration of an RGB camera mounted in front of a microscope with strong color distortion. Appl. Opt..

[29] Sharma, C. *Digital Color Imaging Handbook*. (CRC Press, 2002).

[30] Efron N (1998). Grading scales for contact lens complications. Ophthalmic Physiol. Opt..

[31] Papas EB (2000). Key factors in the subjective and objective assessment of conjunctival erythema. Investig. Ophthalmol. Vis. Sci..

[32] Chong T, Simpson T, Fonn D (2000). The Repeatability of Discrete and Continuous Anterior Segment Grading Scales. Optom. Vis. Sci..

[33] Murphy P, Lau J, Sim M, Woods R (2007). How red is a white eye? Clinical grading of normal conjunctival hyperaemia. Eye.

[34] Foster DH (2011). Color constancy. Vision Res..

