# Author Correction: Comparison of different smartphone cameras to evaluate conjunctival hyperaemia in normal subjects

**DOI:** 10.1038/s41598-020-60004-7

**Published:** 2020-02-19

**Authors:** Carles Otero, Nery García-Porta, Juan Tabernero, Shahina Pardhan

**Affiliations:** Vision and Eye Research Unit, School of Medicine, Anglia Ruskin University, Cambridge, UK

Correction to: *Scientific Reports* 10.1038/s41598-018-37925-5, published online 04 February 2019

The Article contains an error in the Acknowledgements section.

“Dr. García-Porta is supported by the European Union through the Marie-Sklodowska Curie IF program 2016”

should read:

“This project has received funding from the European Union’s Horizon 2020 research and innovation programme under the Marie Skłodowska-Curie grant agreement No 747441”

