# Supplementary material for: Comparison of different smartphone cameras to evaluate conjunctival hyperaemia in normal subjects
Source: Sci Rep. 2019 Feb 4;9:1339. doi: 10.1038/s41598-018-37925-5 (PMC6362079; doi:10.1038/s41598-018-37925-5)
Supplement: Supplementary file 1 — Figure 1S [file 41598_2018_37925_MOESM1_ESM.pdf]

Otero C, García-Porta N, Tabernero J, Pardhan S. Comparison of different smartphone cameras to evaluate conjunctival hyperaemia in normal subjects.

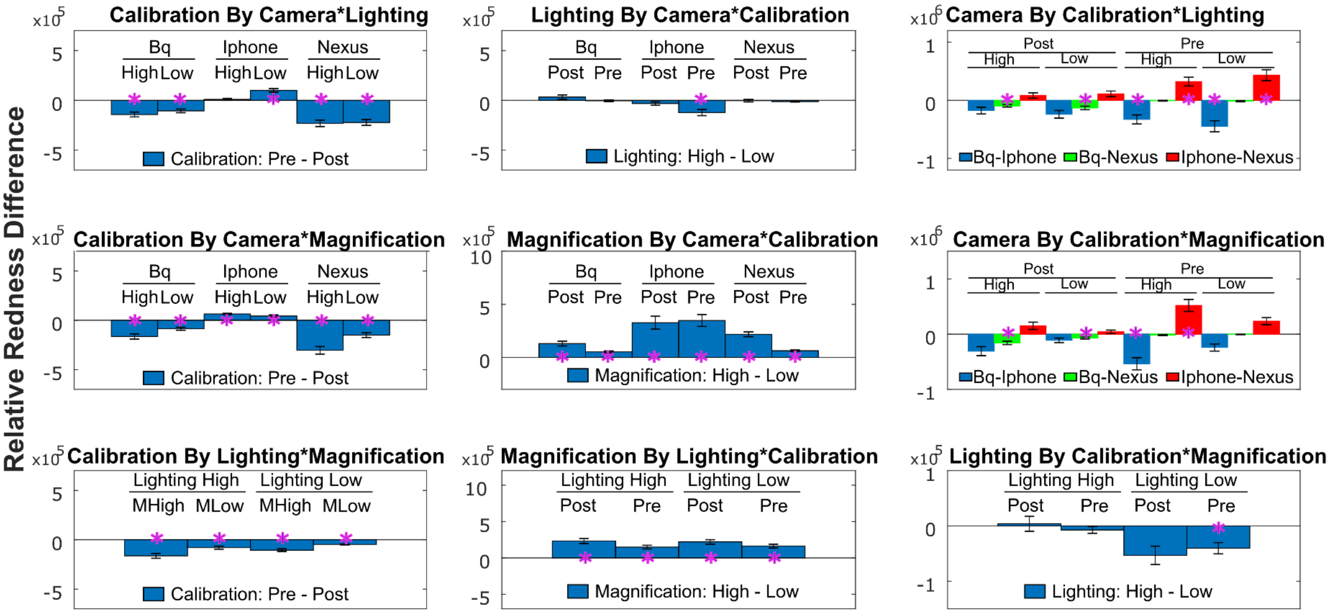

Figure 1S. Error bars for each statistically significant 3-way interaction found in table 3. Purple asterisks indicate statistically significant pairwise comparisons (p<0.05).
